# Morphological Analysis of Several Bamboo Species with Potential Structural Applications

**DOI:** 10.3390/polym13132126

**Published:** 2021-06-28

**Authors:** Asier Elejoste, Alfonso Arevalillo, Nagore Gabilondo, Amaia Butron, Cristina Peña-Rodriguez

**Affiliations:** 1 ‘Materials + Tecnologies’ Research Group, Chemistry and Environmental Engineering Department, Faculty of Engineering, Gipuzkoa, University of the Basque Country (UPV/EHU), Plaza Europa 1, 20018 Donostia, Spain; nagore.gabilondo@ehu.eus; 2 TECNALIA, Basque Research and Technology Alliance (BRTA), Sede Azpeitia, Área Anardi 5, E-20730 Azpeitia-Gipuzkoa, Spain; alfonso.arevalillo@tecnalia.com

**Keywords:** bamboo, Phyllostachys aurea, Arundinaria amabilis, *Dendrocalamus strictus*, morphology

## Abstract

Bamboo constitutes a family of plants that are very promising and interesting as renewable materials for both large and small structure construction. To be used as an alternative to traditional materials; the understanding of its morphology and mechanical behavior is of crucial importance. As the distribution of fibers and vascular bundles differs for each type of bamboo; several bamboo types have been characterized: Phyllostachys aurea (PA), Arundinaria amabilis (AA) and *Dendrocalamus strictus* (DS). Morphological analysis has been performed by optical (OM) and scanning electron microscopy (SEM). Differences in density; surface morphology and wall thickness have been found. In fact; PA and AA have shown a great morphological regularity; while DS presents the greatest thickness; to the point that it can be considered full culm. The plant’s own ducts constitute a very important factor for future impregnations and the optimization of mechanical properties for structure construction.

## 1. Introduction

Due to increasing environmental concerns, the scientific community in the field of materials science is striving to find new renewable structural materials. In this context, bamboo is gaining greater recognition in the marketplace amongst consumers of sustainable and environmentally friendly materials [1,2,3,4,5], representing a greener alternative than the normally employed less sustainable materials. Indeed, in comparison to wood, which is characterized by a long growth cycle [6,7], the compressive strength of bamboo is two times higher compared to concrete and its tensile strength is almost equal to that of steel. Different tests have shown that the ultimate tensile strength of bamboo ranges from 140 to 280 N/mm^2^, equal to mild steel [8]. By using bamboo as a construction material, economic and ecological benefits are obtained, as can be seen in the study by Nayak et al. [9], which compared the cost of steel reinforcement with bamboo reinforcement. This study showed that some bamboo species have a tensile strength equivalent to that of mild steel and work very well in bending. It is also an added benefit in earthquake-prone areas due to the energy absorption capacity of bamboo. This study concludes that the use of bamboo as a substitute for steel reinforcement is a good option in low-cost structures, being three times cheaper.

Bagchi et al. [10] conducted several similar studies on the use of bamboo as a construction material to replace standard concrete material. They also came to the conclusion that the relationship between bamboo’s mechanical properties and cost makes it a very good alternative to concrete as an environmentally friendly building material, due to its high strength and low cost [11]. Bamboo regenerates faster and produces less wastage of raw material in pruning. In addition, several authors indicate that bamboo retains larger amounts of carbon than trees, which is crucial for the reduction of the greenhouse effect and the fight against climate change [12,13,14]. Bamboo is included in the Bambusoideae subfamily, with around 90 genera and more than 1200 species. Bamboo grows naturally in all the continents, being especially abundant in Asia, with 65% of the total world production, mainly in China, followed by Central and South America with 28% and Africa with 7%. However, Brazil possesses the widest variety of bamboo species [15,16,17]. Bamboo covers a growth area of almost 38,000 million hectares, approximately 3.2% of the total forest area around the world. Regarding the cultivation of bamboo, it is estimated that there are 22 million hectares around the world with nearly 4000 different uses [18]. The cultivation of bamboo is desirable as it protects against soil erosion, can be grown in acidic land and it is often used to recover degraded areas as it spreads to inhospitable regions where other plants will never survive. However, bamboo is still not well known across Western civilization [12].

As a structural material, bamboo provides both high specific strength and rigidity, and low water absorption compared to wood [19,20]. The specific strength of bamboo is also higher than that of other common structural materials such as wood-based composites, concrete, and steel [21,22]. However, as with other species, bamboo usually presents a hollow culm and, subsequently, mechanical properties vary along the plant, limiting its use in construction as a structural material [23]. Due to its morphological characteristics, it is mainly used in the form of bamboo derivatives, for furniture production, flooring and in construction and civil engineering [24]. Indeed, bamboo has been used as a homogeneous construction material only in the form of laminated boards or complex composite materials, once the initial bamboo culms are destroyed and reprocessed; i.e., the fibers are separated and later put back together artificially [19,25,26,27,28,29,30,31,32]. However, the entire bamboo culm could be satisfactorily used for construction purposes, such as railings, balconies, or small structures to support electrical wiring, taking advantage of the native effective orientation of cellulose fibers. In order to use bamboo in its original form as a structural material, the first step must be to understand its morphology, thus enabling its treatment in a more efficient way.

**Figure 1:**
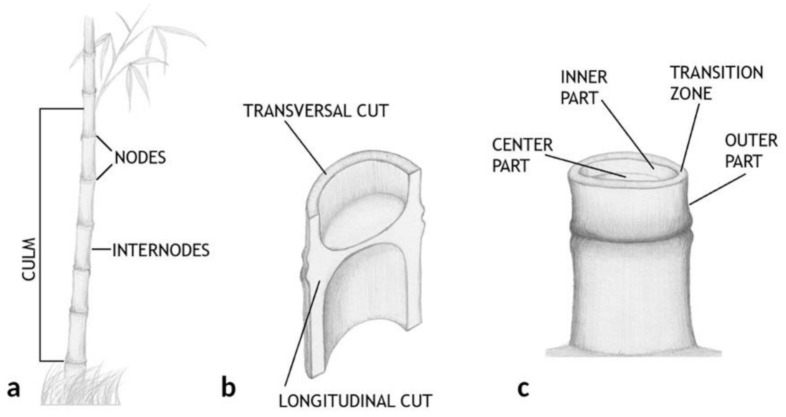
Bamboo scheme: (**a**) view of the plant from the ground to the beginning of the branches, (**b**) longitudinal and transverse sectioned view and (**c**) circular cross section.

The schematic structure of bamboo plants is shown in Figure 1. Bamboo is composed of culms with solid transverse diaphragms or nodes separating different hollow inter-nodal regions (Figure 1a). Unidirectional cellulosic fibers are oriented parallel to the longitudinal axis of the culm (Figure 1b) and embedded in the parenchyma tissue matrix composing the circular cross section (Figure 1c) [12]. This parenchyma tissue matrix lignifies and hardens as the culm matures, leading to increased density and improved mechanical properties. Only a few studies have been carried out on the morphology of bamboo, most of them focused on Guadua or other species not analyzed in this work [1,23]. However, even if the reported studies are mostly related to other properties or practices of bamboo, they usually refer to its morphology [20,24,27,33,34] revealing the necessity of a deeper comparative study in order to better understand the internal structure of the plant and the way to use it.

The knowledge related to the morphology of the plant is required in order to solve the two main limitations of bamboo: the durability of the structure and the heterogeneity of the plant in terms of mechanical properties. One of the proposed strategies is the impregnation of the culm of bamboo with suitable resins. For that, in this work, the fiber and duct distribution has been analyzed for three bamboo species to identify the most suitable impregnation conditions in order to generate materials with efficient transmission of stresses from the matrix to the fibers.

## 2. Materials and Methods

### 2.1. Materials

The most common bamboos used for the manufacture of small structures have been selected for this work. Culms of PA bamboo (*Phyllostachys aurea*), AA bamboo (*Arundinaria amabilis*), and DS bamboo (*Dendrocalamus strictus*) were gently provided by Bambusa. They were impregnated by immersion in borax salts for preservation. The employed materials were young bamboo culms grown after at least six cutting/growing cycles of the plant. The culms obtained in the seventh grow of the bamboo plant can be considered homogeneous in terms of morphological and mechanical properties. PA comes from Anji County, Huzhou, Zhejiang, at an altitude of 200 to 500 m, with an ambient temperature of 18 °C with highs of 28 and lows of −3, humidity of 5.47 ± 0.61%, presenting a density of 863 kg/m^3^ (362 kg/m^3^ if the hollow center is taken into account). AA comes from Anji County, Huzhou, Zhejiang, at an altitude of 200 to 500 m, with an ambient temperature around of 18 °C with highs of 28 and lows of −3, humidity of 5.72 ± 0.15%, presenting a density of 940 kg/m^3^ (497 kg/m^3^ if the hollow center is taken into account). DS comes from Kanchanaburi, Thailand at an altitude of 400 to 600 m with an ambient temperature between 10 and 30 °C and a humidity of 6.6 ± 0.7%, presenting a density of 624 kg/m^3^. For all the study and subsequent tests, the entire culm has been used, as the final objective is the use of the complete culm in structures.

### 2.2. Methods

A Nikon OM E80i microscope was used for optical microscopy (OM) (Nikon Corporation, Japan) characterization both in transmission and reflection modes. The samples were firstly immersed in distilled water, under vacuum for water penetration, and then maintained in boiling water for 1 min. The softened samples were cooled down and cut using a Leica Ultracut R microtome equipped with a diamond knife in rectangular pieces of 0.2 mm in thickness for transmission and 10 mm for reflection.

Scanning electron microscopy (SEM) (Hitachi, Ltd., Chiyoda, Tokyo, Japan) characterization was performed with a Hitachi S-4800 microscope at different magnifications. SEM micrographs of 15 nm gold-coated samples were obtained at 10 kV.

## 3. Results and Discussion

### 3.1. General Morphology of Bamboo

**Figure 2:**
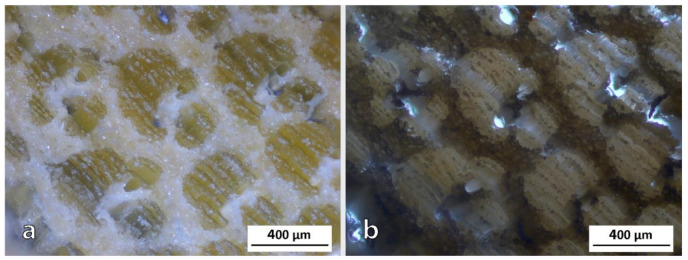
OM micrographs from the cross-section of AA bamboo as obtained by (**a**) reflection and (**b**) transmission modes.

The culm is the part between the roots and branches, which makes up approximately two thirds of the plant (Figure 1a). The general morphology of the bamboo culm was analyzed by OM in both reflection and transmission modes. Images obtained from the cross-section (transversal cut) of AA bamboo culm are shown in Figure 2.

As could be observed, the fibers were more visible in the images obtained in reflection mode (Figure 2a). The fiber packages appear darker due to the loss of light at the bottom of the fibers, whereas the parenchyma appeared bright due to its higher reflection capacity. This image is virtually the negative of the image obtained in transmission (Figure 2b).

**Figure 3:**
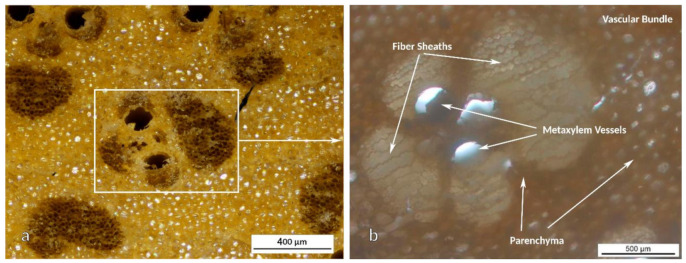
OM micrographs from the cross-section of AA bamboo: (**a**) transversal cut and (**b**) vascular bundle in detail.

The different elements of the culm can be identified with more detail from the OM images shown in Figure 3.

As can be observed, vascular bundles make up the repeating pattern all along the cross-section of the culm (Figure 3b). Those fiber packages or sheaths are responsible for maintaining the shape and structural integrity of the plant. The largest fiber sheath is composed of xylem or protoxylem, whereas the smallest one, always closer to the outer part of the bamboo culm, contains phloem fibers. Vascular bundles are the brightest groupings seen in the image due to the light transmission through the fibers. On the other hand, the darkest areas observed in the image, those between the fiber packages, are small parenchyma cells (Figure 3b). Parenchyma, as one of the so-called fundamental tissues, is involved in a wide variety of functions such as photosynthesis, storage, processing of organic substances and tissue regeneration. Parenchyma appeared darker in this case due to the higher density of the tissue of small-sized cells. In addition, other vascular elements that allow light transmission appear in Figure 3. These refer to the metaxylem vessels, which are the pipes responsible for water and food transport throughout the plant. These ducts are clearly observed in Figure 3a. As is well known, lignin is the aromatic biopolymer responsible for reinforcing the structure of major plants, keeping all plant cells stuck together, which is also the case for bamboo. Moreover, the role of lignin in metaxylem vessels is especially important because as it is not water-soluble; it prevents the metaxylem vessels from collapsing due to the water flow. Lignin also contributes to thermal stability by providing structural rigidity, the lignin content ranges from 18.35% to 25.80% [35,36,37].

In summary, three elements can be differentiated in the inner part of the culm: fibers, ducts or metaxylem vessels, and parenchyma (Figure 3b). The fibers are the structural part of the plant, providing the required strength to stand upright and cope with the external mechanical stress. The ducts ensure the circulation of water and nutrients, while the parenchyma cells participate in food storage. Finally, lignin keeps this network of cells, conduits, and fibers stuck together. The distribution of these elements in terms of percentages and densities depends not only on the species analyzed, but also on the part of the culm section that is being analyzed.

**Figure 4:**
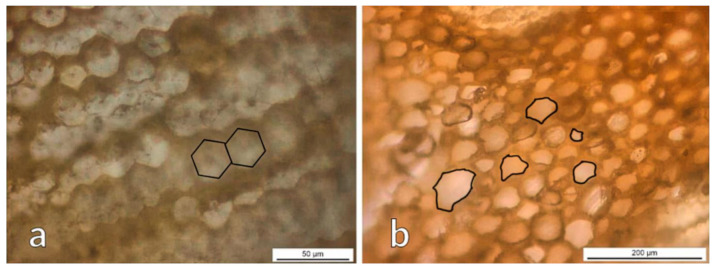
Optical micrographs from the transition zone of AA bamboo: (**a**) transversal cut of fiber sheaths and (**b**) transversal cut of parenchyma.

From the OM images of the transition zone in AA bamboo shown in Figure 4, the appearance of different features can be observed. Fiber sheaths are almost geometrically perfect in shape (Figure 4a), while if parenchyma is analyzed (Figure 4b), cells show different shapes and sizes, grouped in a much more erratic way.

**Figure 5:**
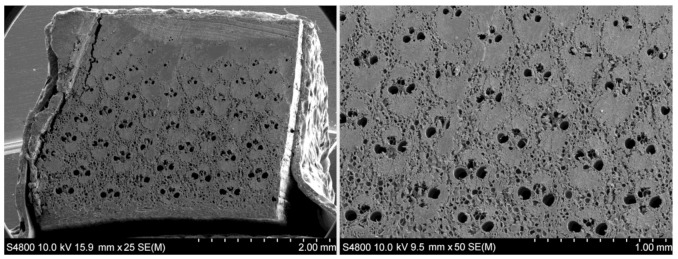
SEM images of transversal cut of PA bamboo with different magnifications.

**Figure 6:**
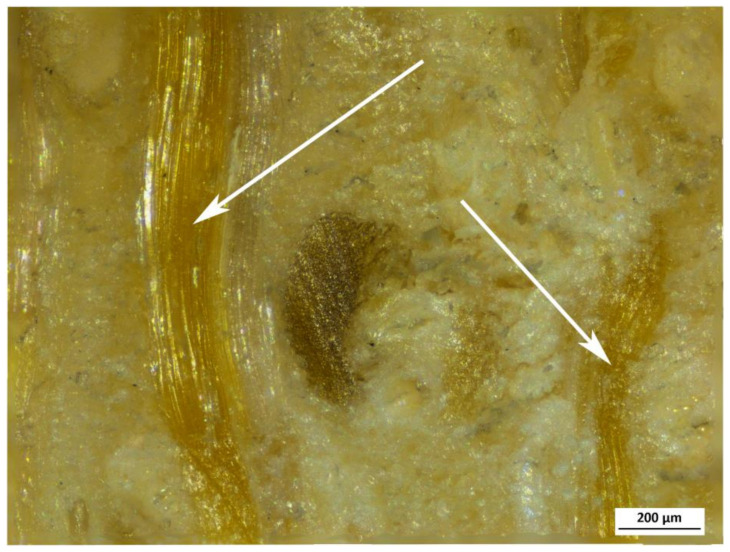
Optical micrograph from the transversal cut in a node of AA bamboo.

As can be appreciated from SEM images of PA bamboo shown in Figure 5, the vascular bundles constitute the pattern that is repeated throughout the transversal section of the bamboo culm. Taking into account that the growth of the plant occurs only apically, the form and distribution of the fibers, metaxylem vessels and ducts of the culm is maintained from the base to the end. However, this pattern is slightly affected near the nodes, where more radial contact appears between the fibers and the ducts, thus giving the plant a greater resistance to torsional stresses [38]. In the internodes, both the ducts and the fibers are axially oriented, while the nodes provide the transverse interconnection. As can be seen in OM micrographs shown in Figure 6, in some nodes, small packages of fibers can be found even in the transverse direction (indicated with arrows). This distribution, in addition to improving the resistance to torsion or combined forces [39,40,41,42], also allows the plant to retain its integrity and makes the culm fracture much more difficult [39,40,43,44]. Thus, the node is the most resistant part of the bamboo, as can be read in the study by Chen [45], which, among the data provided, we can find a tensile strength of 130.93 MPa for internodes and 162.12 MPa for nodes.

**Figure 7:**
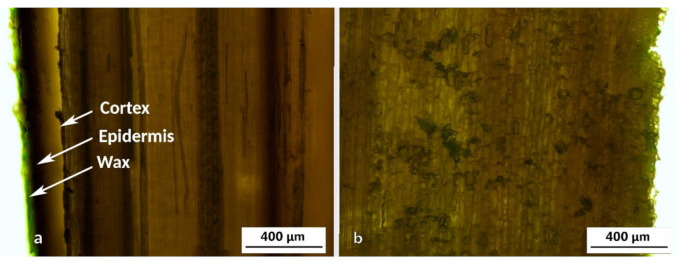
OM micrographs from longitudinal cuts of AA bamboo: (**a**) outer part and (**b**) inner part.

In order to analyze the morphological features close to the limits of the circular section at bamboo culm, longitudinal cuts were also performed, as shown in Figure 7. These areas are difficult to observe in the transverse cuts as they are usually damaged during sample preparation. When cutting the specimens, the softer inner part is deformed by crushing, while the most brittle outer one tends to splinter. In Figure 7a the outer part is clearly visible, composed by the cortex and the epidermis and the wax that protects the culm [38]. In the inner part (Figure 7b), the highest concentration of parenchyma cells is observed along with sclerenchyma cells, which are usually dead cells with thickened and lignified secondary walls [23]. The sclerenchyma, one of the three types of fundamental tissues in plants, is composed of rigid and non-stretchable cells and is generally found in disused parts of plant bodies, such as mature stems [23,46,47]. As can be seen, the straightness of the lines that appear diminishes closer to the inner part, aggravated especially by the disordered accumulation of cells.

**Figure 8:**
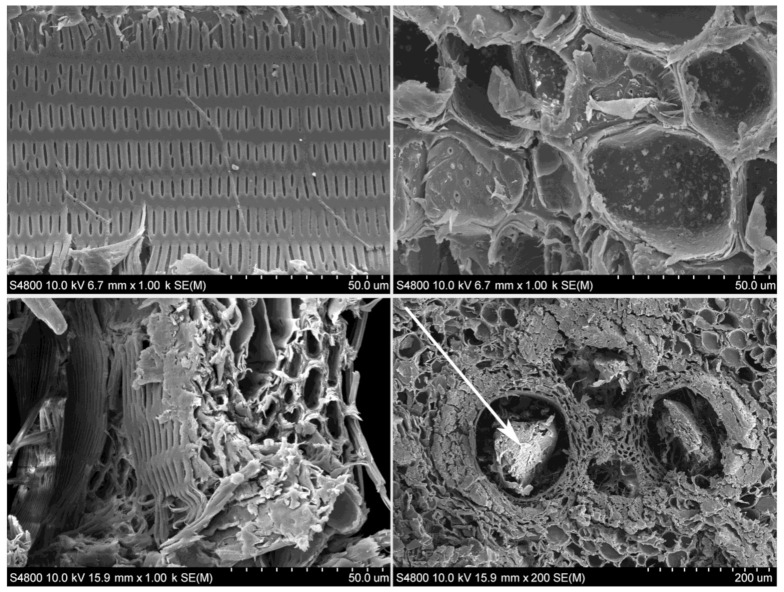
SEM images of (**a**) longitudinal cut of AA, detail of vessel, (**b**) parenchyma cells of AA, (**c**) detail of vessels and cells of AA and (**d**) obstructed vessels of DS.

In the innermost part of the culm, the size of fiber packages gradually decreases to disappear, as can be seen in Figure 7b. The parenchyma cells take on more relevance, with large areas composed exclusively of parenchyma observed. The amount of metaxylem vessel is also increased in this area. This specific way of grouping together the different cells, with the greater importance of fibers outside and food storage inside, turns the bamboo culm into a bar with an extraordinary geometry. For any bar, regardless of the material, mechanical stresses (tensile, compression, torsion, etc.) will concentrate at the outermost part. Therefore, the evolution of species has made most of the fibers concentrated at the area in which they are most needed. This concentration decreases when approaching the inner part, and finally completely disappears. In Figure 5, the complete section of the bamboo wall can be seen. In that image, the ducts distribution can be easily identified. It can be observed that in the outer part of culm, ducts become smaller until they disappear, due to the fact that the vascular bundles are massive and compressed. In the inner part of the culm, they become larger until they disappear, and with them, also the fibers. In this way, with respect to the structural elements of the bamboo culm, the longitudinal fibers of the culm and the lignin keep them compact and provide tensile, compression and flexural strength. Instead, the transversal fibers located in the nodes and the lignin prevent relative longitudinal displacement between fibers, providing torsion strength. In Figure 8, the elements of vascular bundle and parenchyma are shown by SEM images with higher magnification.

Along the whole length of the metaxylem vessel small holes are appreciated, for supplying nutrients to cells (Figure 8a). In the zone of parenchyma cells (Figure 8b), the aforementioned small ducts are also observed, allowing nutrient transmission. The transversal connection between vessels and parenchyma cells is shown in detail in Figure 8c. The liquid flow in the transversal direction is possible due to the interconnection of cells by these small pits (Figure 8b). This is because the horizontal movement of conductive vessels towards the neighboring parenchyma tissue and the fibers occurs solely by diffusion, thus being very slow [38]. Moreover, the circulation through the vessels is reduced when the culm is harvested and matured. Therefore, tyloses and limos produced on the neighboring parenchyma cells move towards the vessels, blocking the conductive cavity [38]. This phenomenon is clearly observable in the bundle of Figure 8d.

### 3.2. Morphological Comparison of Different Species

**Figure 9:**
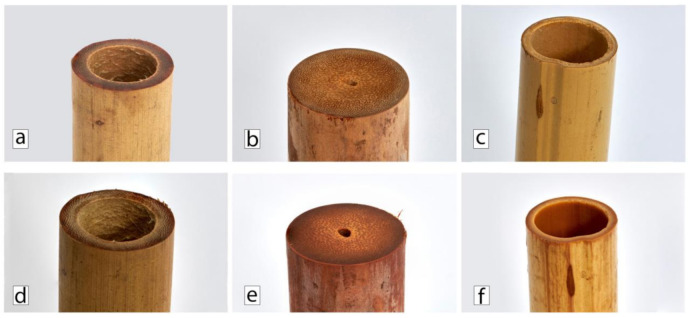
Pictures of AA, DS and PA bamboo culms before ((**a**), (**b**), (**c**), respectively) and after ((**d**), (**e**), (**f**), respectively) water immersion.

Figure 9 shows photographs of AA, DS, and PA bamboo culms both before and after water immersion.

DS bamboo presents a very rough surface with high thickness, whereas PA bamboo shows a perfectly finished thinner surface. There are also differences regarding their wetting behavior after water immersion. Unlike the AA and DS samples, in the case of PA bamboo, the water impregnation is clearly visible in Figure 9.

**Figure 10:**
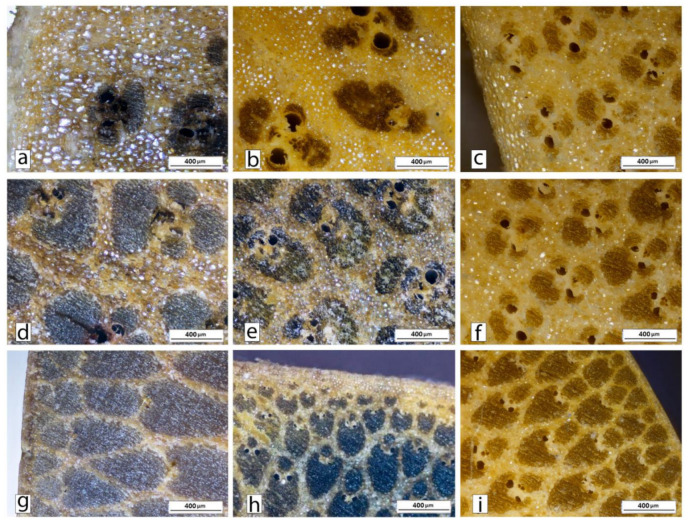
OM images of the inner part of (**a**) AA, (**b**) DS and (**c**) PA species, together with those corresponding to the transition zones of (**d**) AA, (**e**) DS and (**f**) PA species and the corresponding images of the outer parts of (**g**) AA, (**h**) DS and (**i**) PA.

Figure 10 shows the OM images for the inner and outer part of the three different species, together with those of the transition zones. Regarding the inner parts, it can be observed that the central part of DS bamboo (Figure 10b) presents exactly the same morphology as the inner part of the rest of the species. It is not always hollow, even though in the central part almost everything is parenchyma. Small clusters of fibers with their vessels are found, which allow the conduction of nutrients to the cells of the central part. However, on closer observation, it is noticeable that fiber packages in DS bamboo present an erratic orientation. The premise that the smallest bundles will always be closer to the outside of the bamboo culm is not fulfilled in this case. It can be stated that each grouping is facing a different side. On the contrary, as can be seen in Figure 10a,c, both AA and PA maintain the aforementioned structure, following the path discussed throughout the manuscript.

**Figure 11:**
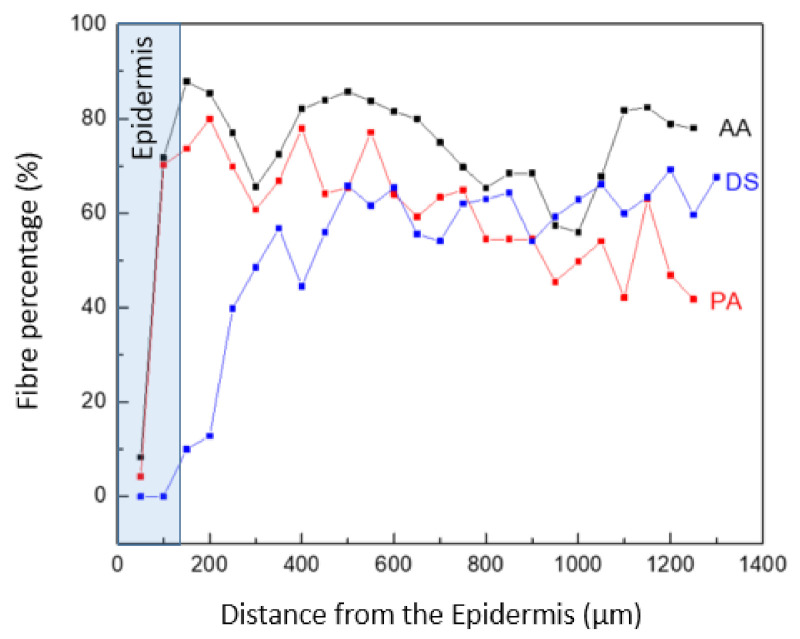
Fiber percentage in the transversal section of the culm of AA, PA, and DS bamboo species. The external zone.

Analyzing the surface area ratio occupied by fibers and parenchyma, differences were found between the three species. The area to be analyzed was the outer part, since it is the most interesting one in terms of mechanical properties. As could be observed in Figure 11, the proportion changes as it goes deeper into the culm. The main difference was found in the epidermis, which is defined in the first 50 µm for PA and AA species, while for DS, it extends beyond 200 µm.

Regarding the inner parts, two other characteristics can be found for DS bamboo that could directly affect the mechanical properties. The first one is the distance between the fiber sheaths. As AA presents lower parenchyma cells density, the fibers are present. On the other hand, in the case of both the AA and DS species, the fiber sheaths appeared to be almost black, while for PA, they were clearer due to higher light reflection, indicating lower fiber density. Furthermore, in the case of PA, the fiber sheaths are much smaller than in the DS and AA species, undoubtedly being the weakest inner part among the three species.

The so-called transition zone between inner and outer zones corresponds to a region of 5 mm close to the skin in the case of DS (Figure 10e), and to the intermediate zone between the outer and inner regions for the rest of the species. This part of DS, compared to that of AA (Figure 10d), shows that the distance between fiber groups is somewhat higher. In addition, more metaxylem ducts are observed but not as much as in PA (Figure 10f).

**Figure 12:**
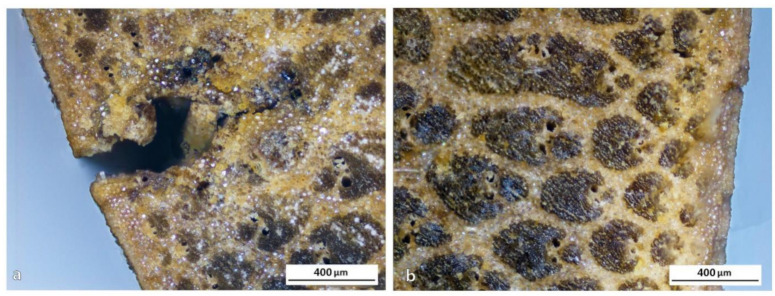
OM images showing defects in DS bamboo: (**a**) skin imperfections and (**b**) morphological irregularity.

**Figure 13:**
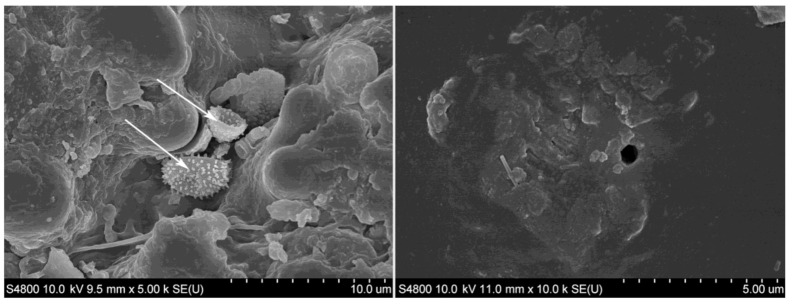
SEM images of (**a**) DS epidermis and (**b**) PA epidermis.

Differences in the metaxylem ducts of AA and DS species can be observed in Figure 10g,h, respectively. In the case of DS, these ducts are present not only in all the external parts but also bordering the epidermis. This fact could allow for a better resin impregnation in the external part, which could lead to a significant improvement of the mechanical properties. However, the cellular appearance of parenchyma could be perfectly noticed in the skin of DS bamboo, indicating that it is not as compact as in the two other cases, consequently being a weaker surface easier to attack. On careful analysis of this surface, many imperfections appeared, such as an erratic distribution of fiber groupings in the center of the culm (Figure 10b), or cracks that can be seen in the OM images of Figure 12. However, this does not seem to be relevant because it is in the center of the trunk and in that area the distribution of fibers would not affect the mechanical properties. The critical point is the presence of imperfections in the outermost area, as seen in the image of Figure 12b. The relevance of these imperfections of DS bamboo culm was confirmed by SEM images of Figure 13, in which pollen and fungi particles located in one of the fissures of the epidermis could be appreciated.

PA bamboo, on the other hand, shows diametrically opposite features (Figure 10i and Figure 13b), with a much more homogeneous and better structured skin. As discussed above, the shape and distribution of the fibers in the PA and AA species is practically the same. However, for PA bamboo, the part in which the fibers have a purely structural use, free of ducts with a minimal surrounding parenchyma, occupies a much smaller area than in AA (Figure 10g,i). Moreover, the metaxylem ducts of PA are abundant close to the epidermis, which will benefit the resin penetration in the external part accentuated by the absorption capacity of this species.

The evaluation of the morphology of different species is essential to assess the homogenization of mechanical properties by impregnation. In fact, it can be deduced that the stabilization of bamboo by impregnation will occur in the longitudinal direction, which differs from wood, in which transversal transport also occurs. This is because radially oriented ducts are present in wood, facilitating liquid flow in the transverse direction. Compared to wood, the percentage of conductive tissue in bamboo is very low, between 5 and 10%, while it is around 70 and 30% in softwoods and hardwoods, respectively [33,38]. It seems that a very efficient impregnation technique will be needed to achieve significant results in terms of mechanical properties.

### 3.3. Morphology, Impregnation and Mechanical Properties

Bamboo presents a well-defined epidermis that represents a drawback for the impregnation process according to European standards by autoclave processes. However, in the case of DS, it could be more likely to achieve this method of impregnation due to irregularities in the epidermis (Figure 13a) but the impermeable layer provided by the bamboo epidermis makes it very difficult for any liquid to penetrate it, and even harder to achieve a minimum of impregnation. To use bamboo as a structural nucleus, as a sandwich technology—that is, covering it with another fiber—it would be necessary to eliminate the epidermis, so that the resins could partly impregnate the bamboo also being in contact with the fibers. However, when bamboo does not require coating, it is convenient to leave the epidermis intact since it provides extraordinary protection for the culm. The most useful solution could be the internal impregnation by longitudinal resin injection with pressure along the ducts of the culm (Figure 8a). In addition, a vacuum can be used to overcome the resistance of small pits present in the interconnection of the parenchyma cells (Figure 8b). In that way, not only longitudinal but also transversal impregnation by diffusion occurs.

The impregnation of the outer area, which contributes most in terms of the mechanical resistance of the culm, is limited because the ducts become closer and disappear (Figure 5), limiting the improvement of mechanical properties by internal impregnation. However, in terms of durability, these ducts’ distribution allows internal impregnation of the inner zone, the weakest for both insects and fungi, which will probably make the culm more durable toward biotic attack. The protection system of plants generating tyloses and limos in the ducts (Figure 8d) to obstruct them when it is cut helps their natural durability. For impregnation, it is not necessarily negative, as the obstruction is not total, being probable that an over-stressing of the impregnation machine in that duct favors the diffusion to the adjacent cells, thus achieving a more complete impregnation.

Bamboo nodes constitute an advantage for both impregnation and mechanical behavior, because they offer a connection between fibers and ducts (as observed in Figure 6). On the one hand, it is good for impregnation, since if some ducts were obstructed in some internode, these ducts would be impregnated through the nodes. Moreover, the transverse contact between fibers improves the mechanical properties in terms of torsional resistance, also helping the plant to maintain the tubular geometry, making it more resistant to bending.

## 4. Conclusions

The morphology of the analyzed bamboo consists mainly of fibers, parenchyma, and ducts. Differences in the morphology of each analyzed type of bamboo indicate that PA and AA bamboos have better defined and regularly distributed morphology compared with DS, whereas AA and DS have a higher density of fibers than PA. The higher protection from biotic and abiotic factors is related to a very regular epidermis, as found with PA bamboo.

Regarding the application and taking into account the fiber and duct distribution, two opposite cases have been found. On the one hand, AA bamboo is the most resistant but the most difficult to be impregnated. On the other hand, PA bamboo seems to be the weakest, but the easiest for impregnation. Therefore, it is also possible to reinforce the outer part, which is structurally the most important part of the plant. Finally, DS bamboo seems to be the one that will be able to have very good impregnation, since not only does it have that capacity in the wall, but also, as this species is solid, it could be completely impregnated. Unfortunately, this bamboo is very heavy and its porosity makes it much heavier, even when impregnated, making it lose the attractive bamboo property of a high strength/weight ratio.

Therefore, the question arises as to which bamboo will be most appropriate for structural use, a question that shall be answered with impregnation and mechanical tests.

Each part of the plant is specialized in one or several functions. Thus, the fibers are responsible for the structural response, while ducts transport the nutrients needed by the plant. The parenchyma, on the other hand, is responsible for storing food and carrying out the fundamental processes of the plant, such as regeneration or photosynthesis.

## Data Availability

The data presented in this study are available on request from the corresponding author.
